# Engineering prostate cancer in vitro: what does it take?

**DOI:** 10.1038/s41388-023-02776-6

**Published:** 2023-07-12

**Authors:** Adriana Buskin, Emma Scott, Ryan Nelson, Luke Gaughan, Craig N. Robson, Rakesh Heer, Anastasia C. Hepburn

**Affiliations:** 1 Newcastle University Centre for Cancer, Translational and Clinical Research Institute, Paul O’Gorman Building, Newcastle University, Newcastle upon Tyne, NE2 4HH UK; 2 Faculty of Medicine, Department of Surgery and Cancer, Imperial College London, Hammersmith Hospital Campus, London, W12 0NN UK

**Keywords:** Prostate cancer, Cancer models

## Abstract

A key challenge in the clinical management and cause of treatment failure of prostate cancer (PCa) is its molecular, cellular and clinical heterogeneity. Modelling systems that fully recapitulate clinical diversity and resistant phenotypes are urgently required for the development of successful personalised PCa therapies. The advent of the three-dimensional (3D) organoid model has revolutionised preclinical cancer research through reflecting heterogeneity and offering genomic and environmental manipulation that has opened up unparalleled opportunities for applications in disease modelling, high-throughput drug screening and precision medicine. Despite these remarkable achievements of organoid technology, several shortcomings in emulating the complex tumor microenvironment and dynamic process of metastasis as well as the epigenome profile limit organoids achieving true in vivo functionality. Technological advances in tissue engineering have enabled the development of innovative tools to facilitate the design of improved 3D cancer models. In this review, we highlight the current in vitro 3D PCa models with a special focus on organoids and discuss engineering approaches to create more physiologically relevant PCa organoid models and maximise their translational relevance that ultimately will help to realise the transformational power of precision medicine.

## Introduction

Prostate cancer (PCa) is the second commonest cancer and the fifth leading cause of cancer death among men worldwide, accounting for 1.4 million new cases and 375,000 deaths in 2020 [1]. At diagnosis, most cancers are localised to the prostate gland (80%) with a minority of patients exhibiting invasion to local pelvic lymph nodes (LNs, 15%) or distant metastasis (5%) [2]. Notably, the five-year survival rate drops from almost 100% for localised disease to only 30% for metastatic PCa. Growth and progression of PCa is dependent on androgens acting through the androgen receptor (AR). For localised PCa, therapeutic options include active surveillance, surgery or radiotherapy with curative intent. For men with advanced PCa, the mainstay treatment is androgen deprivation therapy, but most patients inevitably develop resistance and succumb to lethal metastatic castration-resistant prostate cancer (mCRPC). Despite the availability of multiple classes of agents that delay disease progression and extend survival, including next-generation AR signalling inhibitors (abiraterone acetate, enzalutamide, apalutamide and darolutamide), taxanes (docetaxel, cabazitaxel), radionuclides (radium-223 chloride, lutetium-177-PSMA-617), and poly (ADP-ribose) polymerase inhibitors (olaparib, rucaparib), the development of effective treatments for mCRPC remains an unmet clinical need. A key challenge in the clinical management and cause of therapeutic failure of PCa is its complex heterogeneity. Research to delineate this has been hampered by the lack of biologically relevant preclinical models. Therefore, modelling systems that fully recapitulate the observed clinical diversity and resistant phenotypes are urgently required for the development of successful personalised cancer therapies.

Despite their limitations, current available in vitro, in vivo and ex vivo PCa preclinical models have yielded important insights into our understanding of the pathogenesis of prostate disease. Due to their infinite growth, cost-effectiveness and capacity for high throughput screening and genome editing, two-dimensional (2D) monolayer cancer cell lines have been widely utilised in PCa research [3]. However, they fail to recapitulate three-dimensional (3D) organisation, the tumor microenvironment (TME), cellular interactions with the extracellular matrix (ECM) and the diverse phenotypic and genetic spectrum of PCa. In vivo animal models, including genetically engineered mouse models (GEMMs) and patient-derived xenografts (PDXs), have been indispensable for studying prostate tumorigenesis whilst having the advantage of incorporating a TME [4, 5]. However, GEMMs ultimately do not reflect the complexity and heterogeneity of human cancers. Though PDXs resemble the original tumor characteristics more closely and are able to generate diverse types of PCa, their generation and maintenance are laborious and expensive whilst also compounded by low engraftment efficiencies limiting their application for high-throughput drug screening and genome editing. Ex vivo PCa culture using patient-derived explants or tissue slices does retain the native tissue architecture, TME and endogenous AR signalling, but is reliant on access to freshly resected tissue samples and is only viable short-term [5]. Breakthrough technological advancements led by Hans Clevers in generating 3D organoids now offer tremendous potential for drug discovery and precision medicine [6, 7]. Although organoids are considered as a promising in vitro model system, they are also associated with their own set of limitations for which researchers are actively exploring ways to overcome through bioengineering.

In this review, we outline the current in vitro 3D PCa models with a special focus on organoids and discuss engineering approaches to create more physiologically relevant PCa organoid models and maximise their translational relevance that ultimately will help to realise the transformational power of precision medicine.

## Current in vitro 3D prostate cancer models

### Spheroids

**Fig. 1:**
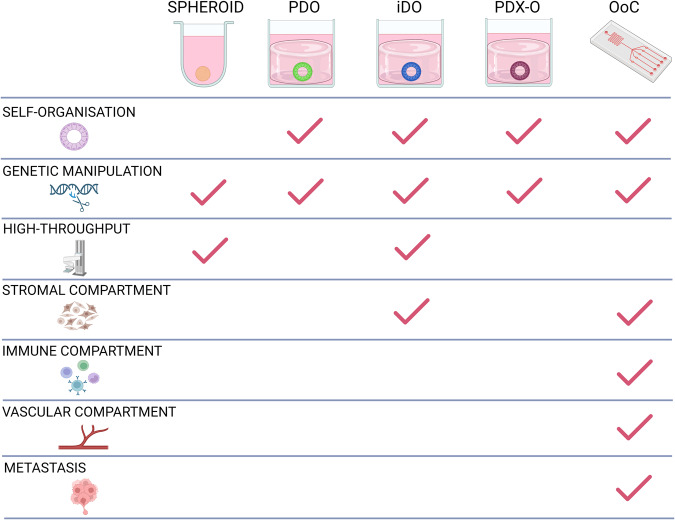
Properties of 3D prostate cancer model systems. Overview of advantages and limitations of each 3D model for studying prostate cancer (Created with BioRender.com).

Spheroids are 3D cell aggregates generated from cancer cell lines and patient-derived cancer cells cultured in suspension using scaffolds, hydrogels or by hanging drop method [8] (Fig. 1). Overcoming some limitations of 2D models, spheroids mimic many in vivo tumor features, such as cell-cell and cell-ECM interactions as well as generating proliferative, metabolic and hypoxic gradients, enabling recapitulation of cellular heterogeneity. Prostate tumor-derived spheroids, ‘prostaspheres’ have also been used to interrogate cancer stem cell-related characteristics in vitro [9]. Though their simple cost-effective generation has favoured their use as models for drug response, spheroids lack organisation and uniformity. Recently, microfluidic systems have further enhanced the development of PCa spheroids. Fluidic systems such as the Microwell Flow Device (MFD) have addressed the challenge of hypoxia-induced necrosis in the core of spheroids, which can mask cellular phenotypes and drug responses [10]. By generating in well laminar flow, PCa spheroids cultured in the MFD exhibited reduced necrotic core formation, enhanced growth and cellular structural integrity whilst also enabled improved modelling of response to chemotherapy. Microfluidics have further facilitated drug screening of biopsy-derived PCa spheroids by enabling self-generating perfusion of nutrients and the formation of repeatable drug concentration gradients [11].

### Patient-derived organoids (PDOs)

Organoids are self-organising, 3D in vitro structures derived from pluripotent stem cells (PSCs) and adult stem cells (ASCs) that faithfully mimic, through provision with biochemical and biophysical cues, the in vivo epithelial architecture, genetics and function of the organ of origin [7]. PSC-derived organoids are established from either induced pluripotent stem cells (iPSCs), generated by reprogramming of adult somatic cells, or embryonic stem cells (ESCs), with both cell sources having the capacity to differentiate into all cell types of the three germ layers (endoderm, mesoderm, and ectoderm), thus enabling the production of both epithelial and non-epithelial lineages. In contrast to PSC-derived organoids that recapitulate in vivo organ development, ASC-derived organoids model adult tissue regeneration. Established directly from healthy or tumour tissue containing the intrinsic ASCs whose differentiation capacity is limited to the cell types of their tissue of origin, ASC-derived organoids represent only the epithelial compartment and are therefore less structurally complex than PSC-derived organoids. Currently, organoids do not fully recapitulate the in vivo microenvironment. Primarily, based on 3D culture conditions previously described for the long-term expansion of organoids from small intestine, organoids have been derived from healthy prostate and advanced PCa tissues [6, 12–14]. Using this culture system, prostate organoids were composed of both basal and luminal cells harbouring multipotent progenitor cells and retained intact AR signalling [14]. Patient-derived organoids (PDOs) established from metastatic and circulating tumor cells can retain the histological and molecular features of the patient tumors and recapitulate the diversity of clinical PCa subtypes, including TMPRSS2-ERG fusion, SPOP mutations, TP53 loss, PTEN loss and CHD1 loss [13]. Drug screening studies using biobanks of PDOs have shown correlation of in vitro drug sensitivities with patient genotype and treatment response, highlighting organoids as a promising tool for precision oncology [15]. Despite their promise, PCa PDOs are associated with low success rates of establishment ( < 20%) mainly due to the same difficulties faced by primary prostate cell culture [13]. Additionally, overgrowth of tumour cells by benign epithelial basal cells present in the biopsy sample has been observed indicating a growth advantage for normal cells in these culture conditions and constitutes a major obstacle for successful PCa organoid establishment [13, 16]. Maintenance of the mature luminal phenotype in culture has also been difficult. Therefore, thorough characterisation of organoid cultures and strategies that promote cancer cell growth over that of normal cells and that also further improve culture conditions for establishment of organoids with the unique characteristics of PCa is necessary.

### iPSC-derived organoids (iDOs)

To overcome the challenges faced by PDOs, iPSCs have enabled the generation of human prostate organoids [17, 18]. Using a coculture approach with inductive urogenital mesenchyme (UGM), prostate iPSC-derived organoids (iDOs) recapitulated the full breadth of prostate epithelial differentiation, including neuroendocrine cells. These organoids formed glandular structures that recapitulated prostate tissue histology and expressed key prostate markers such as AR, prostate specific homeobox protein NKX3.1 and secretory prostate specific antigen. This approach proceeded previous studies demonstrating prostatic differentiation from ESCs, further avoiding the ethical and regulatory challenges associated with the latter [19, 20]. The prostate iDO model presents a promising new tool to study PCa disease and to advance drug testing and personalised medicine. iDOs and genetic manipulation of iPSCs using genome editing technologies such as Clustered Regulatory Interspaced Short Palindromic Repeats (CRISPR)/Cas9 provide an emerging platform for the creation of patient genotypic avatars in vitro that through the generation of organoid biobanks could facilitate the design of powerful drug screening programs leading to future discovery of novel targeted therapeutics. Indeed, oncogenic gene manipulation in iDOs has enabled glioblastoma and pancreatic cancer modelling in vitro [21, 22].

### PDX-derived organoids (PDX-Os)

PDX-derived organoids (PDX-Os) are 3D organoid cultures derived from PDXs. Beshiri et al first reported generation of PDX-Os from the LuCaP PDX model demonstrating their utility for drug testing [15]. PDX-Os retain the genotypic and phenotypic characteristics of the original PDX enabling matched in vivo/in vitro models and development of biobanks. Though this model overcomes some of the limitations of PDXs such as capacity for high-throughput screening and genetic manipulation, its generation does rely on successful engraftment of the original patient sample, with PCa associated with low PDX establishment efficiencies. Additionally, their maintenance in culture long-term is limited and is dependent on the specific organoid growth medium used with optimal conditions needing to be defined for each PDX-O culture [23].

### Organs-on-chips (OoCs)

Organs-on-chips (OoCs) are microsystems designed to recapitulate tissue-specific functions such as cancer metastasis, inflammation, and infection [24]. Human cells or organoids are placed on chips connected by fluidic microchannels reproducing either blood or airflow in a controlled manner to mimic the physiology of the human body. With this technology, it is also possible to construct 3D models of human tumors in vitro, called tumors-on-chips, where the metastatic cascade can be recreated in a precise stepwise manner [25].

### Bioprinting

3D bioprinting is an automated high-throughput system capable to construct multi-layered organoids with tissue architecture in a controlled environment using computer-aided design [26]. Living cells, small molecules and biomaterials are deposited into dishes in a spatial-temporal ordered manner to develop patient-specific cancer models, resembling the heterogeneity of the microenvironment of real tumors. Complex internal tissue structures, including vasculature and stroma, can also be bioprinted to produce different layers of cells such as normal tissue-specific cells, connective tissues and cancer cells [27].

## Engineering strategies to enhance the 3D prostate cancer organoid model

**Fig. 2:**
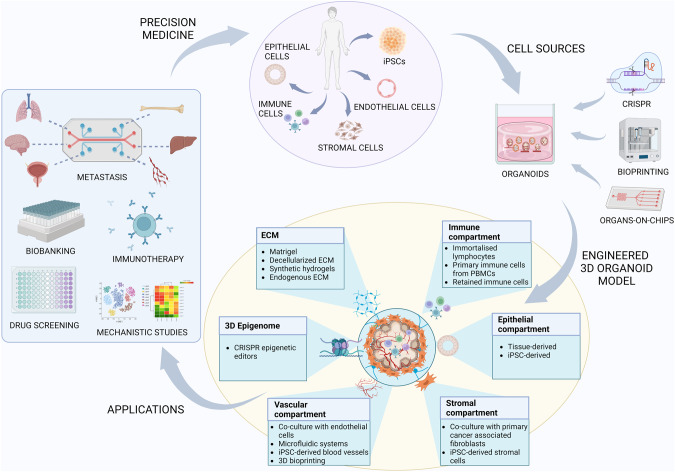
Engineering strategies to enhance the 3D prostate cancer organoid model for precision medicine. A summary outlining the cell sources and bioengineering tools and approaches that may facilitate development of a 3D prostate cancer organoid model that fully recapitulates the tumor microenvironment and overcomes limitations of the current preclinical systems. Such an engineered cancer model would allow more translationally relevant mechanistic studies, high-throughput drug screening, assessment of immunotherapies, generation of biobanks and recreation of the metastatic process and sites for the development of personalised medicines. (Created with BioRender.com).

Current in vitro PCa organoid models still lack an intact TME, which undergoes complex changes as the tumor develops that hugely impact primary tumor growth, metastasis and treatment response. The prostate TME consists of stromal cells, immune cells, vascular cells and also extracellular matrix (ECM) proteins that interact with the tumor cells through a complex network of multiple soluble factors and signalling pathways [9]. Therefore, an in vitro culture system that incorporates all these cell types needs to be developed to fully recapitulate and gain a deeper understanding of these intricate cell-cell and cell-ECM interactions. Additionally, recreating the epigenetic mechanisms involved in PCa initiation and progression will further provide a more comprehensive model for the development of novel therapies. We discuss bioengineering approaches that may facilitate recapitulation of these key features governing tumor dynamics to develop a 3D PCa organoid model suitable for clinical translation (Fig. 2).

### Engineering the extracellular matrix (ECM)

In vitro 3D systems are often cultured in biologically derived matrices that support cell growth, proliferation, and differentiation. These matrices, termed hydrogels, are 3D networks of hydrophilic polymers that can absorb large amounts of fluids. Due to their permeability, composition and density, hydrogels resemble living tissues and have a strong effect on cell activity and fate [28].

Matrigel, an ECM-based hydrogel, purified from Engelbreth-Holm-Swarm (EHS) mouse sarcoma, has been a popular choice to support 3D organoid growth, especially after the seminal work carried out by Yoshiki Sasai showing the formation of optic cups and stratified neuro retina from ESCs cultured in media supplemented with matrigel and differentiation factors [29]. Studies by Hans Clevers also demonstrated the generation of intestinal organoids from ASCs on matrigel [30], followed by the production of organoids from other tissues, including liver [31], colon [32], pancreas [33] and stomach [34].

Despite its versatility, matrigel is overly complex and undefined. More than 1800 unique proteins have been identified by proteomic analysis, including laminin, collagen, entactin, perlecan and multiple growth factors [35]. The batch-to-batch variability of matrigel impacts on the reproducibility of in vitro systems and makes the identification of factors necessary for organoid formation and function very challenging [36]. Despite the huge advancement in the PCa field, the variability and reproducibility of matrigel-based systems need to be addressed. Matrigel’s mechanic properties, such as elasticity, pore size, creep and stress-relaxation which cannot be easily dissociated from its chemical cues [37], are also variable [38, 39], and its animal origin also hinders clinical transplantation of organoids due to risk of immune responses triggered by possible xenogeneic contaminants in the samples [36].

Given these limitations, other matrices have been developed as alternatives to matrigel. Decellularized ECM from several human organs have been shown to support 3D cultures [40–42]. However, this is limited by donor availability and the preparation of decellularized ECM is difficult, leading to lack of definition and batch-to-batch variability [43, 44]. The use of biomacromolecules with defined composition, such as collagen and fibrin, provide an alternative to reduce variability in organoid systems, although they may lack necessary chemical cues for organoid formation which needs to be considered when supplementing the media [45, 46].

In this space, synthetic hydrogels have emerged as a solution for controlled organoid generation by enabling manipulation of physical and chemical matrix properties. Polyethylene glycol (PEG)-based hydrogels can be adapted to control and enhance stemness and differentiation of organoids, whilst their viscoelasticity has been shown to govern organoid growth and shape [47, 48]. In a recent publication, PEG-based hydrogels engineered with either fibronectin or collagen-derived peptides were used to grow PCa organoids. The organoids in the collagen enriched matrix had enhanced growth compared with organoids in other hydrogels, mimicking cell proliferation of tumor tissues [49]. Despite all efforts to develop tuneable hydrogels, synthetic matrices remain less efficient than matrigel for most organoid cultures.

Organoids can also be made from suspended 3D cell aggregates in ultra-low-attachment plates, coated with hydrophobic hydrogels, to prevent cell attachment [50]. Plates are centrifuged to enable the formation of aggregates by cell-cell interactions and subsequent spheroid production, which can be differentiated into organoids [51]. A recent study compared the potential of brain organoid development in the presence and absence of exogenous ECM. Whilst it was demonstrated that exogenous ECM accelerates tissue polarization and formation of neuroepithelial architecture in a few days, this study also showed that unexposed cultures relying only on endogenous production of ECM, gradually acquired tissue polarisation and refined rearrangement of neuroepithelial structures over an extended period of time, demonstrating self-sustainability of organoid formation by self-organization and endogenous processes [52].

These examples, amongst others, demonstrate the need for better matrices, tailored for specific in vitro applications. The knowledge of biomaterials can also be used to provide more control over in vitro models, as well as raising questions about the indiscriminate use of exogenous ECM for organoid cultures.

### Engineering the tumor-stroma microenvironment

Reciprocal epithelial-stromal interactions are not only fundamental to prostate organogenesis and function but also central to prostate carcinogenesis and progression. Key differences exist in stromal cells between the normal prostate and prostatic tumors. The healthy prostate consists primarily of smooth muscle cells, whereas prostate tumors tend to predominantly contain fibroblasts and activated myofibroblasts forming a reactive stroma responsible for the deposition of ECM. There is now a growing body of evidence that cancer-associated fibroblasts (CAFs) are key drivers of PCa progression [53, 54]. CAFs can remain in a pro-proliferative wound healing state and secrete pro-proliferative factors which drive uncontrolled epithelial cell proliferation. CAF populations are highly heterogeneous with up to 6 distinct subpopulations being identified in human PCa and many studies are ongoing to elucidate the role of CAF subsets in prostate disease pathogenesis [55, 56]. To support studies into the roles of CAFs in PCa, and given key differences in human and murine pathophysiology, it is important that we develop human models which accurately represent the heterogeneous and dynamic stromal compartment of prostate tumors.

Cell populations which make up prostatic stromal compartments are highly plastic and responsive to their surroundings. Selecting the correct culture media, tissue culture plasticware, ECM and nutrients is especially important when culturing them. All of these factors have been shown to alter stromal cell phenotypes in culture, and as such they may not fully recapitulate the stromal compartment of patients. This, taken alongside the heterogeneous nature of PCa has made modelling the stromal compartment difficult.

Early prostate organoid and co-culture studies highlighted an important role for stromal cells in promoting epithelial organisation which is crucial for the formation of prostate organoids [57, 58]. Recent reports have corroborated this finding, using co-cultured stromal cells with epithelial organoid cultures. Richards et al. showed that the co-culture with primary prostate stromal cells helped to improve organoid viability, increased organoid formation efficiency, helped to promote a morphologically complex branching phenotype through cell-cell interactions and the secretion of important growth factors [59]. The authors also demonstrated that due to cell-cell interactions, direct co-culture methods are superior to those utilising transwell inserts. Similarly, co-cultures including various cell types to improve maturation of organoids have been utilised in other tissues [60]. Mesenchymal stem cells (MSCs), for example, increased lung organoid formation through promoting alveolar differentiation and factor secretion, whilst paracrine signals from MSCs and human umbilical vein endothelial cells (HUVECs) promoted hepatocyte maturation in liver organoids [61, 62].

Taking a different approach to modelling the prostate stroma in 3D in vitro cultures, the prostate iDO model demonstrated that the iPSCs also have ability to generate and maintain a stromal compartment [63]. In the future this model may prove useful for studying mesenchymal-epithelial interactions, with the ability to account for inter-patient heterogeneity. It is evident that the stromal population plays a key role in PCa pathogenesis and developing models which successfully recapitulate stromal compartments could have real translation value.

### Engineering the tumor immune microenvironment (TIME)

Prostate tumors are commonly thought to be immunologically ‘cold’ characterised by low numbers of infiltrating cytotoxic lymphocytes and an abundance of suppressive myeloid populations [64, 65]. The advent of single-cell technologies has facilitated a greater understanding of the immune context of PCa with distinct clusters found including mononuclear phagocytes (monocytes, dendritic cells and macrophages), T cells, NK cells, B cells and mast cells [65, 66].

Due to the immunosuppressive and poorly immunogenic nature of the prostate TIME, immune checkpoint blockade has failed to elicit a robust anti-tumor response in most PCa patients [67, 68]. Though studies in mice have been critical to our understanding of the TIME and provided key discoveries on immune checkpoints, these models are limited by physiological, genetic, and transcriptomic differences in inflammatory and immunological pathways [69]. To improve our understanding of the mechanisms which dictate immunotherapy responses, there is a need for humanised models which capture the complexities of studying the immune system and its interactions with tumor cells in vitro.

Much of the current work investigating the interplay between tumor cells and immune cells have utilised simple 2D culture models. Often these studies have focussed on a specific immune cell type either cultured in conditioned media from PCa cell lines or directly co-cultured with PCa cells. To model the immune compartment, studies have used immortalised lymphocyte lines, such as Jurkat cells, or primary immune cells isolated from peripheral blood mononuclear cells (PBMCs) isolated from the blood of healthy human donors [70]. These 2D models have been used to some effect to model tumor-immune crosstalk and potential immunotherapeutic applications. Lee et al. used PBMC-derived T cells to demonstrate the utility of engineering chimeric antigen receptor T (CAR-T) cells targeting CEACAM5 to induce cytotoxicity in neuroendocrine PCa cell lines [71]. To model multiple immune cell types in vitro, Yamaguchi et al. developed a co-culture system using PCa cell lines cultured with CAR-T cells alongside polarised PBMC derived macrophages [72]. The authors showed that M2 polarised macrophages can attenuate CAR-T activity in vitro, demonstrating the importance of including multiple immune compartments in in vitro models. Although they are useful in the early development of ideas underpinning new immunomodulatory approaches, these models are limited in their immunological assessment of therapies due to their failure to fully recapitulate the heterogenous TIME.

Studies on solid TIMEs have utilised a range of 3D systems including PDOs, PDEs and microfluidic systems [73]. When studying the interplay between immune cells and non-immune compartments in each of these systems researchers must decide whether to study resident immune cells within the tissue/organoid or to look towards 3D co-culture experiments. Whilst PDOs and explant cultures have the potential to retain host-resident immune cells, these immune cells often have poor viability, resulting in a restricted timeframe for meaningful immunomodulatory studies. This is because culture conditions have widely focussed on maintenance of the epithelial and stromal compartments and have been optimised to that end. It is then difficult to define optimal culture conditions for resident immune populations which are not to the detriment of non-immune compartments. Studies utilising the resident immune populations are important for deciphering the role of the non-immune compartments in modulating the TIME. They also provide important platforms for screening for markers of susceptibility or resistance to immunotherapeutic efforts. Both of which will be critical for understanding human mechanisms of resistance to immunotherapies, and how tumor targeting therapies, such as taxanes, can be used to sensitise tumors to immune checkpoint inhibitors.

To overcome time constraints when studying retained immune compartments, co-cultures with PBMC-derived cells, or tissue isolated immune cells, which are autologous to the PDO, have been established. These models are particularly important for the study of immune subsets, such as T cells, which need to be Human leukocyte antigens (HLA)-matched to the epithelial compartment. There are fewer complications when studying innate immune subsets, such as mononuclear phagocytes (MNPs), where HLA-matching is not necessary, and cells from unmatched donors are sufficient. These models allow users to optimise culture conditions for an immune population of interest, and to study their function in the presence of tumor cells in vitro. Whilst allowing for longer studies focussed on immune cell phenotype and interaction, these studies are limited when attempting to study many immune cell compartments together. Optimal culture conditions to maintain immune cell viability or polarisation may include immunostimulatory conditions which may not allow for the co-culture of certain immune cell types together.

Currently, studies utilising these more complex tumor-immune systems for the study of PCa are lacking, but insights can be drawn from research into other solid malignancies. Immune profiling studies on non-small cell lung cancer (NSCLC), renal cancer and melanoma PDOs demonstrated retention of CD8+ and CD4 + T cells, CD14/CD69+ macrophages, NK and NKT cells and B cells [74]. These studies were successfully used to model T cell activation in response to an anti-PD-1 therapeutic antibody, demonstrating the utility of the system for immune checkpoint blockade studies. Human pancreatic organoids have been successfully cultured with CAFs and human peripheral T cells where the authors observed T cell infiltration in a PDO dependant manner [73, 75]. Building on this, studies on NSCLC and colon organoids showed that matched PBMC cultures are dynamic, and respond to matched PDOs, resulting in expansion of the CD8+ anti-tumor T cell population in culture [76]. This ability to modulate retained immune compartments in vitro has also been modelled in tissue explant cultures of NSCLC. The authors revealed a metabolic immune shift in response to pembrolizumab (anti-PD-1) in NSCLC tissue slices accompanied by tissue damage. Interpatient heterogeneity in response to pembrolizumab treatment was also observed [77]. Findings from these studies highlight the potential in these systems to model patient responses to therapies and their effect on the TIME in vitro. Developing similar systems targeted towards modelling the prostate TIME would almost certainly accelerate the development of personalised immunotherapeutic approaches for PCa patients.

### Engineering the tumor vasculature

The lack of organoid vascularisation still remains a major challenge in the field and limits a broader application and impact. The incorporation of a functional vasculature in organoid systems to provide a sufficient supply of oxygen and nutrients as well as metabolic wastes disposal is essential for their growth and maturation [78]. Fluid flow through the vessels further enables cellular communication and distribution of key biochemical factors and drugs, whilst mechanical fluid forces also play a critical role in vasculature development. Currently, organoids rely entirely on passive diffusion of nutrients, gases and metabolites through the culture medium eventually limiting their size and proliferation and resulting in the formation of an inner necrotic core. Therefore, remodelling perfusable vascular networks, that also remain stable in time, is necessary for the lifespan and true recapitulation of the in vivo complexity and functionality of organoids. Moreover, modelling of the tumor-vascular interactions will increase insights into metastasis, immune cell trafficking and delivery of anticancer therapeutics. To date, functional organoid vascularisation has only been achieved by in vivo transplantation, demonstrating the ability of organoids to become vascularised given the right environment [79].

Strategies being explored to overcome current limitations and incorporate a vasculature within organoids include introduction of endothelial cells (ECs), microfluidic organ-on-chip devices and 3D microfabrication technologies [78]. The most common method to vascularise organoids is co-culture with ECs, such as HUVECs and iPSC-derived ECs, either through coating onto organoids or a co-differentiation approach. Particularly, use of iPSC-derived ECs from the same source of the organoid would greatly favour organotypic vasculature formation, though organ-specific EC differentiation still needs further research. Furthermore, human blood vessel organoids derived from iPSCs were shown to contain ECs and pericytes that self-assembled into capillary networks in vitro and also formed functional blood vessels in vivo [80]. To overcome the obstacle of nutrient supply and emulate mechanobiological features, microfluidic strategies have evolved utilising 3D hydrogel scaffolds to build perfusable vascular networks enabling precise spatiotemporal control. Progress in 3D bioprinting has also facilitated a breakthrough in fabrication of vascular networks [81]. Recently, culture of iPSC-derived kidney organoids in a 3D printed, gelbrin matrix coated millifluidic chip under high fluidic shear stress exhibited enhanced vascularisation during nephrogenesis [82]. However, the development of matrices and culture conditions that support both organoid growth and the engineered vasculature still remains an ongoing challenge. Continued advancements in bioengineering and bioprinting technologies are necessary for the development of fully vascularised organoid models.

### Engineering the metastatic tumor microenvironment

Currently for men with metastatic disease there are limited treatment options that target the metastases themselves. PCa primarily metastasises to the lymph nodes (LNs) and bone, but also to liver, lungs and brain [83]. However, organ tropism of PCa metastasis remains poorly understood. Models that recreate this multistep process of PCa metastasis and TME of the metastatic site that evolves in parallel are essential to identify treatment strategies for patients at this stage of the disease. As well as developing representative models of the metastatic sites, models that represent patients most likely to metastasise are also necessary.

#### Lymph nodes

LNs are the first site of spread for most cancers, including prostate. During metastasis, PCa cells escape the primary tumor and invade the sentinel LN parenchyma before further dissemination through the bloodstream. Developing models of the LN and lymphatic system is essential to further understand the key factors and processes that lead to metastatic spread with potential for identification of early biomarkers for high-risk patients.

Models of LNs have included ex vivo tissue slices, microfluidic chips and engineered tissues, including organoids [84]. Due to the fluidic nature of the lymphatic system, increasing efforts have been made using microfluidic platforms to recreate the LN microenvironment with several LN-on-chips being generated [85]. Using microfabrication techniques, Shanti et al, developed a microfluidic system that incorporated immune cell types embedded in 3D hydrogels allowing for interrogation of immune evasion mechanisms and also demonstrated the fluid flow was comparable to that of native LN lymphatic fluid enabling further probing of dissemination through the lymphatic system [86].

#### Bone

PCa patients with bone metastasis harbour osteoblast lesions and suffer from skeletal related events including multifocal pain, fractures and hypercalcemia. Invasion of PCa cells in the bone results in disruption of normal homeostasis of bone formation and resorption. The bone is composed of osteoblasts, osteocytes, osteoclasts and osteogenic cells and their interaction with PCa cells leads to a ‘vicious cycle’’ of bone matrix remodelling whereby through secretion of chemokines and growth factors osteoblasts stimulate formation of bone with poorly organised collagen fibrils and reduced mechanical strength [87].

The bone microenvironment comprises bone marrow mesenchymal and hematopoietic stem cells, ECM, and a vascular network [83]. In vitro 2D co-cultures of PCa cell lines and bone cells have provided some initial insights into the changes they undergo and crosstalk during the metastatic process. Though GEMMs and PDXs have provided most of our knowledge about the PCa metastatic microenvironment, in vivo models that lead to bone metastasis are limited despite it being the predominant metastatic site [88]. Additionally, the mouse and human bone TME differs with respect to ECM, bone marrow composition and the vascular and immune compartments. In vitro 3D co-cultures incorporating scaffolds offer improved modelling of bone ECM which not only has a role in the structural function of the bone but also provides growth factors and chemokines supporting colonisation of PCa cells in the TME and cell-cell interactions. Furthermore, 3D printing now enables development of scaffolds that more closely mimic native bone properties, such as stiffness and pore size [89]. A 3D engineered microtissue model of PCa osteoblastic metastasis allowed determination of the effects of antiandrogen therapies [90]. More recently, microfluidic platforms offer the opportunity to model the dynamic process of metastasis from the primary site to the distant organ, whilst also allowing incorporation of a vascular network as constructed for the study of breast cancer bone metastasis [91]. Though a promising tool, bone organoids have not been developed yet. However, iPSCs can generate osteoblasts and osteoclasts underscoring their potential utility for engineering not only functional but also personalised bone models [92].

#### Liver

Compared to other metastatic sites for PCa, liver metastasis occurs at similar frequencies to lung metastasis and is associated with the worst clinical outcomes for PCa patients [93]. Disseminated tumor cells from the primary site invade the circulatory system and once extravasated from hepatic blood vessels then colonise the liver [94]. Multiple cellular components, including parenchymal hepatocytes and non-parenchymal cells such as Kupffer cells, sinusoidal endothelial cells, bone marrow-derived immune cells, fibroblasts and hepatic stellate cells, together with cell adhesion molecules, chemokines and collagen proteins, provide the unique complex microenvironment that renders the liver hospitable to disseminated tumor cells and influence decisions to undergo apoptosis, dormancy or aggressive outgrowth [95].

Hepatocyte organoids have been successfully generated, whilst iPSC technology has enabled development of multilineage liver organoids incorporating many non-parenchymal cells and also a vasculature [96, 97]. Microfluidic platforms to model liver metastasis have been developed, such as the liver-on-a-chip (liver-chip) two channel device devised by Kim and colleagues to reconstruct the liver microenvironment through promoting the growth of sinusoidal endothelial cells, fibroblasts and hepatocytes in order to study metastasis from breast cancer [98]. Though this system could be improved by incorporation of additional non-parechymal cells and an in vivo-like engineered ECM, it has scope for utility for the study of PCa liver metastasis and development of new therapeutic strategies.

#### Lung

PCa lung metastases are also associated with shorter overall survival times compared to bone and LN metastases and are less responsive to treatments [93]. There has been limited progress in modelling lung metastasis and therefore opportunities to advance this field exist. Characteristics that play an important role in the lung as a site for metastatic cancer and should be modelled include vascularisation with dual blood supply, abundance of immunosuppressive cells and angiogenesis [99]. PCa organoids derived from lung metastasis have been reported but efficiency has been hampered by overgrowth of normal lung epithelial cells [13, 16]. Furthermore, 3D multicellular organoid models, such as the “primitive lung-in-a-dish (PLiD)” that incorporates lung epithelial, fibroblasts, lymphatic and blood vessel endothelial cells, offer an opportunity to study lung metastases [100]. PLiD mimicked the lung microenvironment with formation of air sac–like structures and production of lung surfactant protein, whilst growth with colon and ovarian cancer cells enabled interrogation of lung colonization and therapy response indicating potential utility for precision medicine.

#### Brain

Though metastasis to the brain is uncommon and usually detected post-mortem, development of such models is still vital for translational utility. To metastasize to the brain, PCa cells must first cross the blood-brain barrier before disseminating into the brain parenchyma where interactions with microglial and astroglia cells lead to seeding of lesions [101]. Given the complexity of the human brain, PSC-derived brain organoids have been successfully generated and vascularised [102]. Though not yet developed for PCa, such organoids have been used to interrogate metastasis to the brain in other cancers, such as small cell lung cancer [103]. Microfluidic systems have enabled replication of the blood-brain barrier on chips which have been used to develop multi-organ-on chip devices to study metastasis to the brain and identify therapeutic targets, providing scope for PCa [104].

### Modelling the 3D epigenome

Over the last decade, advances in next-generation sequencing technologies have markedly improved our understanding of the PCa genome, transcriptome and also epigenome. Epigenetic alterations, such as DNA methylation and histone modification, affect the accessibility of DNA and chromatin structure and can influence gene expression. It is now widely reported that during initiation and progression of PCa, large-scale epigenetic changes occur, though how to recapitulate these in 3D in vitro models pose challenges for researchers. Targeted epigenome editing technologies hold promise for manipulation of the 3D genome.

Aberrant DNA methylation has been identified across many cancers, including PCa, with distinct methylation patterns reported in benign and tumor tissues [105]. Hypermethylation at specific gene promoters, including adenomatous polyposis coli, *APC*, Ras-associated domain family member 2, *RASSF2*, and glutathione-S-transferase, *GSTP1*, have been consistently demonstrated to be robust biomarkers in detecting PCa [106]. In addition, DNA methylation changes have been shown to define molecularly distinct subsets of primary PCa, ERG-fusion positive and SPOP mutant [107]. Following this, studies have looked to identify key changes in DNA methylation that allow disease to progress to clinically challenging mCRPC. Zhao *et al* uncovered the DNA methylation landscape of mCRPC, highlighting differences with early-stage PCa [108]. Interestingly, PDOs from both early and advanced PCa maintained epigenetic concordance with the original primary tumor [109, 110]. Recapitulation of DNA methylation changes in 3D in vitro models requires highly specific targeted epigenome editing tools. DNA methyltransferases, such as Dnmt1 and Dnmt3, fused to nuclease-inactivated CRISPR/Cas9 (dCas9) have been successfully used for targeted DNA methylation [111, 112]. Similarly, changes in histone modifications have been seen during PCa progression [113]. Dysregulation of histone demethylases and histone methyltransferases has been reported to be associated with acquisition of a more aggressive phenotype and drug resistance [114, 115]. Targeted manipulation using dCas9 fused to histone-modifying enzymes is also a potential approach for modelling these modifications. Use of such programmable epigenetic editors for modelling of the 3D epigenome could provide better insights into the role of epigenetic alterations in PCa [116].

Emerging evidence suggests that changes in metabolite levels may modulate the activity of chromatin-modifying enzymes [117]. Therefore, when modelling the epigenomic landscape in vitro, the influence culture conditions may have on epigenetics should be considered. The use of glutamine in culture media, for example, can modulate histone demethylation through its conversion to the tricarboxylic acid cycle intermediate-α-ketoglutarate, a key cofactor for the Jumonji-domain containing histone demethylases and in turn impact tumor growth and therapeutic response [118]. Similarly, histone methyltransferases require S-adenosylmethionine, an intermediate metabolite generated from methionine in the one-carbon metabolism pathway, to methylate histones, subsequently modulating transcription of dedifferentiation genes and cancer-associated genes [117].

### Recommendations for the future of prostate cancer research

**Fig. 3:**
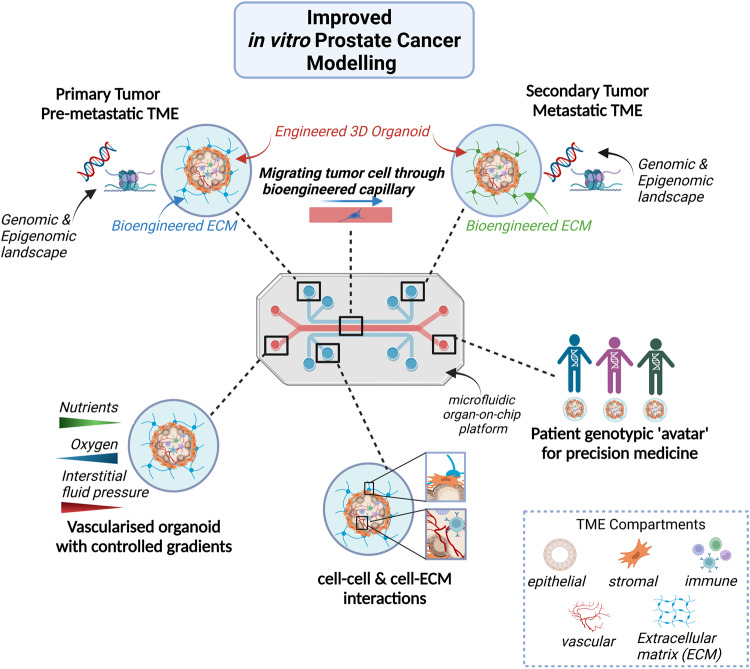
Recommendations for the future of prostate cancer research. A co-culture system integrating 3D engineered organoids and an organ-on-chip platform to incorporate the multiple cell types of the TME would allow development of a more physiologically relevant PCa model amenable for clinical translation and precision medicine. (Created with BioRender.com).

To maximise the translational relevance and applicability of the current in vitro PCa organoid model, its limitations need to be addressed by engineering approaches (Fig. 3):

- Co-culture systems integrating organoids and OcC engineering to incorporate the multiple cell types of the TME would allow development of a more physiologically relevant PCa model amenable for clinical translation.
- Design and use of defined synthetic ECM-based matrices, made from tuneable biomaterials that recapitulate the dimensionality and biochemical and biomechanical properties of tumor tissues and are also tailored for specific in vitro applications, will enable the spatiotemporal and shape control of organoid growth, long-term expansion of PCa organoids, increase reproducibility and provide experimental control.
- Further advancement of the prostate iDO model, with a focus on the generation and maintenance of the stromal compartment will be invaluable for studying PCa epithelial-mesenchymal interactions.
- Development of more complex 3D models utilising the retained immune compartment or matched PBMC cultures in PDO systems should be a priority to study the immunomodulatory capacity of therapies for PCa.
- To overcome the obstacle of nutrient supply and emulate mechanobiological features, such as fluid shear stress and hydrodynamic pressure, microfluidic strategies utilising 3D bioprinting and biomaterial-based matrices should be undertaken to build perfusable vascular networks enabling precise spatiotemporal control and modelling of the tumor-vascular interactions that will allow insights into metastasis, immune cell trafficking and delivery of anticancer therapeutics.
- To model metastasis, biomaterial-based microfluid platforms incorporating both the primary and secondary tumors, vasculature and immune compartment should be developed to bioengineer the pre-metastatic and metastatic niche, creating a ‘metastasis-on-chip’ system for investigation of the biology of organ-specific metastasis and screening of personalized organ-specific therapeutics.
- Use of targeted editing technologies would allow modelling of the 3D PCa epigenome.

## Conclusion

PCa is difficult to treat due to its complex heterogeneity which poses a challenge for current preclinical models to recapitulate and a roadblock in development of effective treatments. The crucial breakthrough in 3D organoid culture has now provided a platform to model cancer heterogeneity in vitro and an opportunity for the clinical translation of precision medicine. Despite this immense progress, current organoid culture approaches have not achieved true in vivo-like function, in turn preventing a broader impact. However, progress in tissue engineering using co-cultures, biomaterials, 3D bioprinting techniques and microfluidic devices is enabling the design of more physiologically relevant engineered 3D models. New engineering approaches are addressing recreation of the dynamic and heterogeneous TME and metastatic process through incorporating the various cellular compartments, developing designer ECMs and integrating biophysical and biochemical parameters. Overcoming organoid size and phenotypic variability also needs addressing to also enable reaching their full potential for high-throughput drug screening. Furthermore, genome and epigenome editing technologies hold promise for manipulation of the 3D genome to recreate the alterations seen in PCa. Integration of these engineering strategies and technologies would revolutionise the PCa organoid model and accelerate clinical translation of precision medicine treatments for PCa patients.
